# Progress and Needs of Dentistry

**Published:** 1893-03

**Authors:** J. Taft

**Affiliations:** Cincinnati, O.


### THE DENTAL REGISTER.

VOL. XLVII.] MARCH, 1893. [No. 3.

### COMMUNICATIONS.

### Progress and Needs of Dentistry.

BY J. TAFT, D.D.S., M.D., OF CINCINNATI, O.

Address of the Chairman, delivered before the Section of Oral and Dental Surgery, at the Forty-third Annual Meeting of the American Medical Association, held at Detroit, Mich., June, 1892.

A backward glance for a year or two upon what has been accomplished affords ground for encouragement.

While, as is the case in almost every department of human occupation, there is a dark and a bright side, aspects discouraging and encouraging, yet it is quite apparent to the unbiased and faithful observer, that the dark side is becoming diminished in extent and lessened in intensity, and the bright is extending in all directions and assuming an increasing brightness. Discouragements are fading and encouragements growing.

I will for a brief time endeavor to direct attention to a few things that seem to afford warrant for this statement. If we look to the mere practical matters of our profession it is quite apparent that never before was there such an intense interest and earnestness shown as at the present time; never were practical methods, processes, instruments, appliances and materials examined, criticised and tested as to-day. It is no longer sufficient for a few to pass judgment in respect to any of these things, with the expectation that such decision will be accepted without question or challenge. While it is true that now more than ever before dentists are willing and even anxious to witness illustrations and demonstrations, it is equally true that every one, especially of the more progressive sort, must verify by his own tests and experience, that which he has witnessed at the hands of others; and as higher attainments have been made in the knowledge of underlying principles, these tests and examinations are more and more exacting and severe, thus bringing the greater probabilities of approximation to the truth.

There is a growing community of interest in all that pertains to the principles and practice of dentistry, in as much as almost the entire profession have given liberally of that which they possess, however it may have come to them.

There is a feeling of intolerance in the profession towards those who hold or retain from others anything new or valuable that may by any means have come into their possession. This principle is clearly presented in the Code of Ethics.

The opinions and views entertained in regard to the education and training of those who are to constitute the dentists of the future are undergoing rapid and radical changes, more pronounced than ever before. The enquiry comes from the pen or lips of every interested one—what can be done to avoid the errors and deficiencies of the past, and to make more thorough, complete and successful, the educational efforts that are now in operation, as well as those that may in the future be inaugurated?

Such questions are indicative of an earnest desire for something better. As a result of these questionings and desires a very pronounced and significant change is now taking place in the educational schemes and methods suggested, which are being tested.

The old, regular didactic lecture method, supplemented by vague and unsystematic clinical demonstrations, are no longer accepted and regarded as properly meeting the requirements; while the power of the lecturer, the living speaker, is esteemed as not less valuable than in the past—indeed, it was never used with greater power and efficiency than at the present time—yet there have been brought into operation with it far better, more systematic, extended and thorough clinical demonstrations, and this not only to the observation of the learner, but he is subjected to the most thorough personal, manual training.

In connection with the lecture method, quizzing, reciprocal questioning, recitation, frequent writing upon the subjects of the course, and essays upon subjects assigned, together with class discussions, are being utilized to the great advantage of the learners.

No educational institutions, especially those of more than an elementary character, can assume to be well equipped for their work without a good library and museum. The library should contain, as fully as possible, the entire literature of all subjects and branches taught in any given institution.

Dental colleges should by no means be an exception in this respect. It is gratifying to know that the dental colleges of the country are turning attention to this important subject. Within the last two years more attention has been given to this question than ever before. Museums and appliances for illustrative teaching are commanding more attention and being more and more utilized. The number of dental schools has been much increased within the last two years, and most of these have established high requirements. It is a fact of great significance that the larger proportion of dental colleges recently organized have been in connection with medical colleges or universities; and some of the older dental colleges have sought and obtained alliances with universities. It is a noteworthy fact that in most cases of such alliance the suggestion has originated within the medical schools, or with the authorities of universities. This fact considered in connection with another, viz: that the first dental college ever organized sought an humble connection with an established medical college, and was not only denied, but rudely repulsed, and with the statement made that the thought of such an alliance could not for one moment be entertained.

The favor with which dental colleges are now received by medical and other institutions is prophetic of great things for the future; it is an appreciation of dentistry by the medical profession that is not properly esteemed by the majority of dentists. Physicians have come to recognize dentistry as a department of the great healing profession, and that for the proper performance of its duties and responsibilities the groundwork of general medicine should be possessed by the dentists.

The increase in the number of dental schools is questioned, and even severely criticised by some; but that there is a surplus of thoroughly efficient schools will hardly be affirmed by any. Quite a number of the schools now in operation should raise their standard of requirements and teaching, or others should be established that would conform to the requirements of the times.

A great and important step was taken at the beginning of the current scholastic year, in the extension of the time required for graduation from two to three terms of not less than five months each. This was done in compliance with resolutions passed by the National Association of Dental Faculties, as follows:

"*Resolved*, That attendance upon three full regular courses of not less than five months each, in separate years, shall be required before examination for graduation."

It was further resolved:

"That we agree to adopt a graded course of instruction and an intermediate examination between each course, which course of instruction and examination shall be conducted as the faculties of the different colleges represented in this Association may deem proper."

This Association has been and is one of the most potent factors in promoting and maintaining the progress and welfare of the profession. Its influence is uplifting, and is in a good degree effective in securing advanced methods of work and harmonious co-operation. During the last two years there has been a larger attendance at the dental colleges of the country than ever before, influenced somewhat, no doubt, by the more extended and strict requirements which came in force October 1, 1891.

A regular systematic course of college instruction and training is now recognized as the only proper method of entering the practice of the profession, and the recent legal enactments regulating the practice of dentistry have been formed with this idea prominently in view. No one who has not had such preparation will be acceptable in the more cultivated and refined communities. In the principal dental societies of the country graduation is a requisite for membership. This is a recognition of the desirability and value of thorough, systematic, scholastic, professional training. This, of course, looks to a higher standard of professional attainments and excellence. There is in all our educational institutions a general disposition to extend the curriculum—the scheme of study and work. Branches that have from the beginning formed a part of the course are now more thoroughly and largely studied, and new subjects are being introduced from time to time, especially those that can be made tributary to the needs of a professional education.

Preliminary education now receives more attention than ever before. This subject received an impulse from the following action of the Association of Dental Faculties:

"*Resolved*, That a preliminary examination be required for entrance to our Dental colleges. Such requirements shall include a good English education. In case of any applicant failing to pass satisfactory preliminary examination, the other colleges of this association shall be informed of the fact.

"*Resolved*, that a candidate for matriculation who presents a diploma from a literary institution, or other satisfactory evidence of literary qualifications, shall be admitted without further examination.

"*Resolved*, That we agree to adopt a graded course of instruction and an intermediate examination between each course, which course of instruction and examination shall be conducted as the faculties of the different colleges represented in this Association may deem proper.

*Resolved*, That after June, 1893, the yearly course of study shall not be less than seven months, two of which may be attendance upon clinical instruction in the infirmary of the school, now known as the intermediate or infirmary course."

This action of the Association was after due consideration passed by more than two-thirds of the members present, and now all the colleges (about thirty in number) represented in this Association conform to the requirements of these resolutions, which is regarded by all as a marked step in the line of progress.

Reference is often made to the rapid increase in the numbers entering our dental schools, and apprehension is expressed lest there should be an excess in the ranks of the profession—more than a supply for the demand. It is well for those who entertain such an idea to consider that this is, from this time onward, to be the only method of entering the practice of dentistry. The office pupilage road is about closed and will remain so. Again, those who graduate from our colleges do not all remain, but from ten to fifteen per cent., recognizing their want of natural ability, or finding some more lucrative and inviting field of occupation, leave the practice of dentistry and enter a more (to them) acceptable service. Again, there are many in the profession who, though they have been in practice for many years, have fallen behind and are not able to cope with well equipped and thoroughly educated dentists of to-day; and they are constantly dropping out and leaving their fields to others better qualified. And as the public become more intelligent and better informed in regard to the value and importance of the teeth, and learn that they are a necessity to the welfare and best interest of the animal economy, and understand fully the capability and resources of dental science and art, the demand will be made, and must be answered, for the best service possible. This growth in intelligence on the part of the people is fully keeping abreast with, if not outstripping, the boasted progress of dental skill and art. It is the bold assertion of one who has given much thought to the subject, that "There are not enough well-equipped dentists in the United States to properly cleanse and keep clean the teeth of all the people of the country who need such service." This statement leaves out of view all other treatments and operations upon the teeth and mouth. Whether or not this statement is literally true, as has been stated, we cannot affirm that there are more well-equipped practitioners in our country than are needed; of the inferior variety there probably is an excess.

By reference to the graduations of the various dental colleges of the country for the year beginning June 1, 1891, and ending June 1, 1892, it is shown that from thirty of the leading colleges the degree of D.D.S. has been conferred upon 1,430 persons, about

10 per cent. of whom are foreigners and will go to other countries, thus leaving the number for this country 1,287. If 10 per cent. of these drop out in a short time, it would leave as the net increase 1,160, which will hardly more than supply the decrease, and this leaves out of the estimate the increase in the country's population. The number of matriculates during the last year in these thirty schools was 2,727; deducting from these the graduates leaves 1,397 from which to form the senior and junior classes of the coming year. This view of the subject, if correct, indicates to us clearly that there is not now, nor is there likely to be in the near future, an excess in the numbers of dental practitioners in this country. And this is without question true in regard to those who possess a thorough preparation. Of this class there probably never will be an excess—more than the public welfare requires.

The progress of any department for the future depends upon the free response to certain requisites. Some of these may here be mentioned.

First in regard to our dental schools. Notwithstanding the improvements and progress that have been made, much yet requires to be done. More thorough work should be required upon most of the ordinary branches of the curriculum. In the past the time has been altogether insufficient, and acknowledgedly so, for the proper accomplishment of the work required. To meet this necessity either longer terms or an additional number should be required. The student's work from the beginning to the end of his course should be continuous. It is a mistake that the student is permitted to follow his special study and work for four to six months in the year and to remain idle, so far as this work is concerned, the balance of the year. He should be made to realize that it is for his interest in some way or other to follow his course of instruction, study, and work continuously from the beginning until his graduation. The time required for the completion of a course will depend upon the extent of that course, the natural ability, industry and energy of the student. It was formerly supposed that two five-months' courses were quite sufficient. This idea now, however, is scarcely entertained by any one; the more extended the course, the greater number of branches embraced, the longer time will be required for the completion of the work upon them. A thorough consideration of this branch of the subject is an important need of the times.

The preliminary qualification and preparation of those who propose to enter upon a course of instruction for dental practice, is one of far greater import than is usually supposed. Heretofore the practice has been that almost any one, regardless of his natural or acquired attainments, has been allowed to enter upon a course of study and work preparatory for dental practice. The evils resulting from this course have been quite apparent. It is impossible to make proper preparation without a right beginning. For the right accomplishment of a dental course a trained mind is requisite, one accustomed to systematic thought and study, and well equipped in the knowledge of the branches of a liberal education. In addition to this, there should be certain natural qualifications and fitness for the work proposed. Rarely does any one present himself for entrance to a college without consulting a practitioner and endeavoring to learn something as to what will be required of him. In all such cases the practitioner should exercise due discrimination and refuse to recommend or advise the unfit to enter upon a course of study. The subject of giving advice to intending students should be thoroughly considered and advisedly exercised by every practitioner. A course of this kind would very much aid our colleges in determining whom to accept or reject. Most colleges now require of those who apply for entrance certificates or testimonials in regard to character and good standing. This, however, is not enough. These certificates are given by good citizens in any walk of life, but it would be well if every student who applies for entrance to a college would bring a statement from a well-qualified dentist, giving his views as to the fitness of the person to enter upon such a course. It would be well if all colleges made such a requirement. It would then become incumbent upon every intending student to consult a good dentist and obtain his advice in reference to his capability. In the preparation of those who are to enter the ranks of the profession in the future it is important that more attention be given than in the past to a broad, liberal education and culture. The great mass of those who in the years gone by have entered the profession have been limited in this respect. A breadth and depth of culture gives power and strength. It makes one better in his profession, gives him a better standing and influence with his fellows with whom he comes in contact and mingles. It would be better, perhaps, as a rule, to require preparatory to entering upon a course of medicine or dental study a classical education, and the tendency evidently is in this direction. A course of not less than one year of private pupilage would be a good initiative for the student, under a well qualified, faithful teacher; but this is so rare that to make it a requirement would avail little or nothing.

But the members of the profession, though they may not be able to take or give proper instruction to private pupils, can be of immense service in an advisory way. Indeed, the profession is quite as responsible for the character of the graduates sent out, as the colleges themselves. A student when applying to a college may be masked, and even remain masked during his course of instruction, so that the authorities of the institution may not know his real character at all; but this view is very seldom the case with the neighbors, friends and associates of the young man. They know him without his mask, and they should see to it that at least the colleges are not imposed upon in this way.

We may not now criticise the past; its work has been done and cannot be recalled; it was accomplished under circumstances and with a light very different from our environments of to-day. Perhaps the best possible was then accomplished, but the demands of the present are of a higher order, and far more exacting than hitherto.

In respect to our colleges permit a suggestion which if carried out would place all upon a higher and more independent plane. As colleges at first, and for many years, were organized, they were dependent for support wholly upon the fees of those who patronized them. The income then was regulated by the number of those in attendance; the larger the number, the greater the income. By-and-by this came to be so fully recognized that a different arrangement was sought, and has in some institutions been carried out, either by endowment of the schools or by some other provision for their support independent of the income from students. A full and complete connection by dental colleges with a university or with endowed medical colleges would seem to be about the only way of escape from this unfortunate condition. It is true that in the future dental colleges may be endowed as such. Nothing of the kind has as yet been attempted so far as I am aware. Endowments for dental colleges have been said to be the great present need for dental colleges. How shall it best be accomplished?

And now a thought or two in reference to this organization which so closely allies us to the general medical profession. The very fact of this Section is an evidence of encouraging progress. It gives to dentistry a position which it did not occupy before; it brings upon its members, and upon the members of the dental profession, an added responsibility; it brings its practitioners into closer alliance with the members of other departments of the healing fraternity. It is prophetic of progress for the future and is a stimulus to those who rightly appreciate this inclination to go forward and make higher and higher attainments, and to render themselves worthy of the high trust placed upon them.

An attempt to measure and estimate the condition and status and progress of dentistry as it stands to-day compared with the past might with entire propriety embrace both the art and science—the practical and theoretical—and not only this but the influences and forces that are operative in promoting its development and growth. In what is now proposed little more than a brief reference will be made to the art side of the subject; the intent is to note more especially some of the forces that are now operative for the further upbuilding and establishment of that department of the healing and restoring art with which we are the more immediately connected.

